# Investigating microvascular outcomes with ischemic preconditioning and passive stretch

**DOI:** 10.14814/phy2.70474

**Published:** 2025-08-01

**Authors:** Sarah A. Fenn, Ward C. Dobbs, Maxwell K. Walker, Britton C. Scheuermann, Jacob T. Caldwell

**Affiliations:** ^1^ Exercise and Sport Science Department University of Wisconsin‐La Crosse La Crosse Wisconsin USA; ^2^ School of Health Sciences Kansas State University Manhattan Kansas USA

**Keywords:** cardiovascular, ischemia, oxidative function, post occlusive reactive hyperemia

## Abstract

This study examined the effects of intermittent passive stretching (PS) and ischemic preconditioning (IPC) on microvascular responsiveness and muscle oxidative capacity. We hypothesized that PS would lead to greater improvements in vascular function and oxidative capacity than IPC. Eighteen healthy male and female participants completed a randomized crossover design, undergoing post‐occlusive reactive hyperemia (PORH) and oxidative capacity testing. PS involved bilateral foot splinting to moderately stretch the gastrocnemius and soleus, while IPC used bilateral thigh cuff inflation. Each intervention consisted of four cycles of 5‐minute “on” and 5‐min “off” periods. A two‐way repeated measures ANOVA assessed condition by time interactions, and intraclass correlation coefficient evaluated the absolute agreement in resaturation rates. A significant main effect of time was observed for resaturation rates (*p* < 0.01). Similarly, a main effect of time was found for the recovery rate constant, with increases observed in both the PS group [pre: 0.88 ± 0.23, post: 0.98 ± 0.28 min^−1^] and the IPC group [pre:0.90 ± 0.33, post: 0.97 ± 0.25 min^−1^] (*p* = 0.019). These findings suggest that both methods, when used acutely, increase microvascular responsiveness and oxidative capacity. Given its ease of application, PS may serve as a practical tool for populations unable to engage in traditional exercise, offering a method to promote functional improvements.

## 1 INTRODUCTION

Ischemic preconditioning (IPC) is a well‐established technique involving intermittent vascular occlusion to protect the myocardium and peripheral vasculature from ischemic injury (Addison et al., 2003; Jones et al., 2014). Near‐infrared spectroscopy (NIRS) has been used to assess muscle microvasculature responses to IPC, revealing adaptations such as reduced desaturation rates during occlusion (Ambrozic et al., 2013; Cortez et al., 2011). In addition, IPC has been shown to enhance the muscle oxidative response to exercise, demonstrated by accelerated muscle deoxygenation kinetics (Kido et al., 2015) and increased skeletal muscle oxidative capacity compared to control conditions (Jeffries et al., 2018). These physiological changes may underlie observed improvements in muscular endurance following IPC (Tanaka et al., 2016). However, despite promising effects in healthy individuals, IPC has produced inconsistent results in improving total walking distance among individuals with peripheral artery disease (Delagarde et al., 2015; Saes et al., 2013). This variability highlights the need to explore alternative or adjunct strategies that may yield more practical and sustainable long‐term adaptations (Caldwell et al., 2023; Ce et al., 2022; McCully, 2010; O'Brien & Jacobs, 2021; Przyklenk & Whittaker, 2011).

Intermittent PS is a physiological stimulus that involves stretching target muscles (e.g., calf, quadriceps, and hamstrings) using a device and is easily performed with minimal cost and equipment (Hotta et al., 2013). PS induces muscle deoxygenation like IPC but with the added benefit of activating muscle metabolism (Kerris et al., 2019; McCully, 2010). This elevated metabolism and shear rate may contribute to the unique benefits of PS, such as enhanced glucose uptake (Kerris et al., 2019), increased endothelial function (Bisconti et al., 2020; Ce et al., 2022; Hotta et al., 2013, 2018), and reductions in vasomotor tone (Wong & Figueroa, 2014). However, while PS consistently improves macrovascular function in postmenopausal females and individuals with peripheral arterial disease (Hotta et al., 2019; Wong & Figueroa, 2014), acute effects on microvascular responsiveness and oxidative capacity remain incompletely understood. This study aimed to investigate acute intermittent IPC and PS by measuring microvascular responsiveness and muscle oxidative capacity. We hypothesized that PS would increase microvascular reperfusion and muscle oxidative capacity more effectively than IPC.

## 2 METHODS

### 2.1 Participants

Eighteen healthy, recreationally active, college‐age (21 ± 1 years) women (*n* = 9) and men (*n* = 9) volunteered to participate in the current investigation (Table 1). Participants reported to the laboratory after a minimum 3‐h fast and were asked to avoid heavy exercise, alcohol, and caffeine for 24 h prior to data collection. Females self‐reported their menstrual cycle and were subsequently tested during the early follicular phase. All experimental procedures and methods were approved by the Institutional Review Board of the University of Wisconsin‐La Crosse and conformed to the standards set forth by the *Declaration of Helsinki*. Informed consent and health history screening for overt diseases (e.g., cardiovascular, metabolic, and renal) took place prior to data collection. Adipose tissue thickness was measured using skinfold calipers prior to the first lab visit; participants with a thickness greater than 1.0 cm were excluded given light penetration depth limitations of the near‐infrared spectrometer (Barstow, 2019). All testing was completed in a temperature‐controlled laboratory (20–22°C).

**TABLE 1:** Baseline characteristics.

Variable	
*n*	18 (9F)
Age, year	21 ± 1
Height, cm	171 ± 9
Mass, kg	73 ± 13
Adipose tissue thickness, cm	0.59 ± 0.24

Resting hemodynamics	Visit 1	Visit 2
Systolic blood pressure, mmHg	122 ± 11	121 ± 10
Diastolic blood pressure, mmHg	66 ± 7	65 ± 8
Pulse Pressure, mmHg	61 ± 10	56 ± 11
Heart rate, bpm	61 ± 10	61 ± 10

### 2.2 Experimental measurements

#### 2.2.1 Intermittent passive stretch

Randomized PS was performed bilaterally in the calf using a night splint to stretch the gastrocnemius and soleus. PS was performed in a seated, upright position with the hips at ~90° and their legs supported on a chair across from them to best maintain their upright body position. At various time points, participants were reminded to keep the knees at 180° and be as upright as possible. To achieve a given level of stretch and provide a level of reference to discomfort, participants were initially stretched to their limit of tolerance (level 5) based on a 1–5 scale (e.g., 1 = no stretch, 2 = mild stretch, 3 = moderate stretch, 4 = intense stretch, and 5 = intolerable stretch) for ~30 s. Next, participants completed four cycles of calf stretching to moderate discomfort (level 3) for 5 min, after which the stretch was released for 5 min. Stretching was performed by dorsiflexing the ankle to level 4 (intense) while researchers held the angle for ~30 s to partially eliminate viscoelastic creep and then set at a moderate level of stretch before Velcro straps were set (Caldwell et al., 2023). The angle was not recorded in the present study.

#### 2.2.2 Ischemic preconditioning

Subjects performed the IPC intervention in a supine position. Rapid inflation and deflation pneumatic cuffs (D.E. Hokanson, Bellevue, WA) were placed bilaterally on the subjects' upper quadriceps and inflated to a suprasystolic pressure (250 mmHg) to fully occlude the lower limbs. The IPC intervention was designed with the same number and duration of cycles as the PS intervention: 4 cycles comprised of 5 min of inflation followed by 5 min of recovery.

#### 2.2.3 NIRS

Microvascular hemoglobin + tissue myoglobin [Heme] were measured with a continuous wave multi‐distance near‐infrared spectrometer (NIRS) probe (Portamon, Artinis) that was placed longitudinally over the lateral head of the right gastrocnemius. Briefly, the NIRS probe consists of a detector fiber bundle, three light‐emitting diodes (LED), and operates at wavelengths of 690 and 830 nm (source detector distance 2.5–4.0 cm). The NIRS device allows for relative changes in oxygenated (oxy‐[Heme]), deoxygenated (deoxy‐[Heme]), and the calculated sum, total (total‐[Heme]) (Barstow, 2019). In addition, the device measures tissue oxygen saturation (StO_2_%). The NIRS probe was allowed to run for at least 2 min prior to the start of data collection. Identification of the muscle belly was identified by a single experienced investigator palpating during muscle contraction and remained in position throughout testing. The NIRS data were collected throughout the protocol at 10 Hz and stored for post hoc analysis in Microsoft Excel.

#### 2.2.4 NIRS—post occlusive reactive hyperemia

This test consisted of a 2‐min baseline, 5‐min occlusion via a rapid cuff inflator (Hokanson, SC12D, Bellevue, WA), and 5‐min post‐occlusion measurement in the supine position. The NIRS parameters were calculated as follows: (1) baseline StO_2_% was calculated as an average over the minute prior to cuff inflation; (2) desaturation rate (slope 1) was quantified as the downward slope from 10 to 60 s following cuff inflation; (3) tissue resaturation rate was quantified as the upward slope of the 10‐s window following cuff release; (4) nadir was calculated as the minimum StO_2_% during occlusion; (5) peak StO_2_% was calculated as the maximal value post cuff release; (6) area under the curve during occlusion, or AUC‐Occlusion, was calculated using the trapezoid rule as an index of the accumulated metabolic stimulus; and (7) AUC‐Overshoot was calculated using the trapezoid rule after cuff deflation and tissue reperfusion (Barstow, 2019; McLay, Fontana, et al., 2016; McLay, Gilbertson, et al., 2016).

#### 2.2.5 Gastrocnemius oxidative capacity

NIRS derived oxidative capacity was measured by performing 1 min of calf exercise at an absolute load of 15 kg in an upright position (i.e., weighted plantarflexion) followed by a series of 14 rapid cuff occlusions, set at 250 mmHg, lasting 5–15 s with similar reperfusion after cuff deflation (Ryan et al., 2012; Southern et al., 2014). For each occlusion, a linear regression was performed over a 4‐s span of data. Data from the first 0.5 s from cuff inflation was omitted as this was the time needed for the Hokanson cuff to inflate, and care was taken to maintain a high tissue saturation at all times (Adami & Rossiter, 2018; Pilotto et al., 2022). All data were transferred into Microsoft Excel to calculate slopes from each cuff inflation. Each slope was plotted as a function of time and fit with the equation *y* (*t*) = End – Delta × *e* ^*−kt*^, where End is the first slope after the cessation of exercise, Delta is the difference between End and slopes calculated at rest, and *k* is the recovery rate constant. This procedure was performed twice with 5 min between measurements and then averaged as the intraclass correlation coefficient of the values between the 5‐min measurements displayed excellent reliability (*r* ≥ 0.90) at all collections. Only slopes with a good fit (*r* ^2^ > 0.85 were used to fit the equation). Initial data points were removed if they dissociated from the curve enough to suggest the point was invalid (Beever et al., 2020).

### 2.3 Experimental design

This study was a randomized crossover design where data collection occurred over two visits with at least a 1‐week interval between them. Upon arrival, the NIRS device was placed on the lateral head of the gastrocnemius, and subjects rested in a supine position for 10 min. Next, blood pressure was taken, and a post‐occlusive reactive hyperemia test (PORH) was performed to assess microvascular function. Next, muscle oxidative capacity of the lateral gastrocnemius was assessed. Following this pretesting, participants completed either the PS or IPC intervention. The opposite intervention was done on the second visit following an identical regimen. Once the intervention was finished, post‐intervention testing initiated after a 10‐minute rest period. Post‐testing mirrored pretesting, starting with a PORH test accompanied by muscle oxidative capacity assessment to evaluate the impact of the intervention.

### 2.4 Statistics

Data were analyzed with a commercially available statistical package (Sigmaplot; version 14.5, Systat software). All data were analyzed with a two‐way (condition × time) repeated measures analysis of variance (ANOVA) with Student–Newman–Keuls post hoc for pairwise comparison. A secondary analysis using a one‐way analysis of covariance (ANCOVA) was performed with a between‐factor of condition (IPC and PS) while including baseline values as a covariate. This approach allowed us to compare posttest outcomes while accounting for baseline individual variability in microvascular responsiveness and oxidative capacity (Vickers & Altman, 2001). To further evaluate the agreement in microvascular responsiveness following the two interventions, the intraclass correlation coefficient (ICC) and the 95% confidence interval (CI) using a two‐way mixed effect model for absolute agreement were calculated between the IPC and PS protocols (Koo & Li, 2016; Shrout & Fleiss, 1979). In addition, the Bland–Altman analysis was used to assess and visualize the bias and limits of agreement between IPC and PS on outcome measurements (Bland & Altman, 1986). The level of significance was set at (*p* < 0.05). All data are presented as means ± standard deviation.

## 3 RESULTS

**FIGURE 1:**
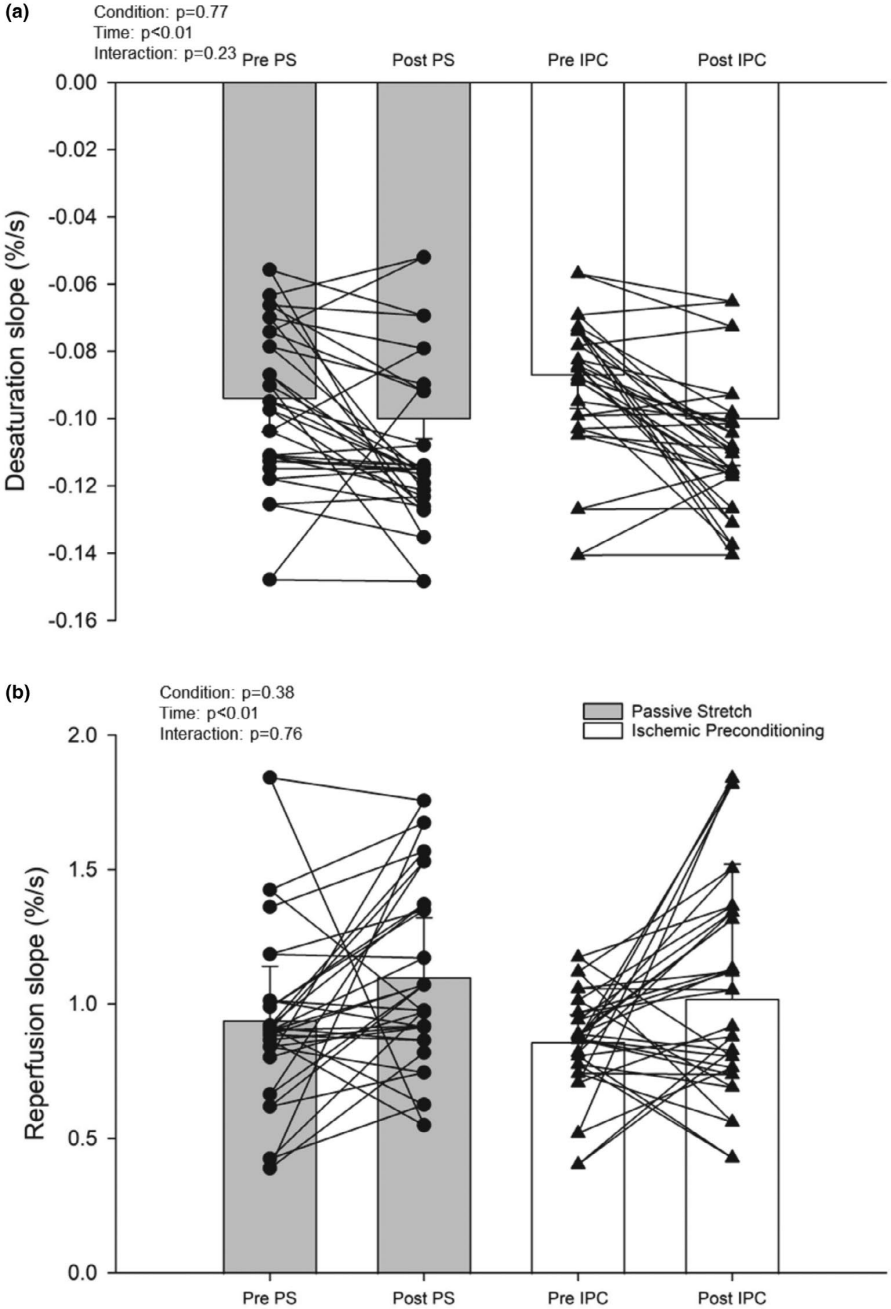
(a) Near‐infrared gastrocnemius tissue oxygen desaturation slopes before and after. (b) Near‐infrared gastrocnemius tissue reperfusion slopes before and after. Means ± SD with two‐way repeated measures ANOVA set at 0.05. Groups include nine females and nine males.

**FIGURE 2:**
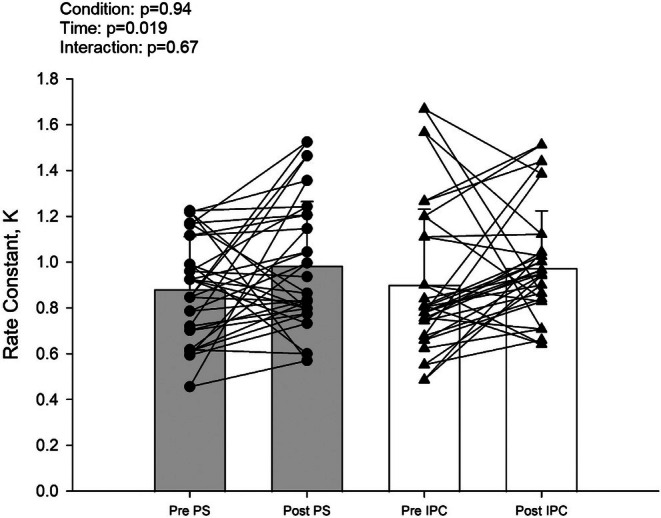
(a) Near‐infrared gastrocnemius rate constants before and after treatments. Means ± SD with two‐way repeated measures ANOVA set at 0.05. Groups include nine females and nine males.

Pretesting values between PS and IPC for baseline StO_2_, nadir, peak, and desaturation rate during occlusion were not significantly different (Table 2). Further, AUC during occlusion was not different, indicating similar metabolic stimuli for reperfusion rates (McLay, Gilbertson, et al., 2016). There were no interactions present for any NIRS variable, but significant main effects of time for baseline, peak, desaturation rate, resaturation rate, and occlusion AUC (Table 2). Desaturation and resaturation rates also showed a main effect of time (Figure 1). No interaction was present for muscle oxidative capacity, but there was a main effect of time (Figure 2). ANCOVA analysis revealed the posttest outcomes were not significantly different between the conditions (resaturation rate [*p* = 0.94] and oxidative capacity [*p* = 0.71]).

**TABLE 2:** Microvascular parameters.

NIRS variables	PS	IPC	*p* Value
			Condition
Baseline			
Baseline, %	71 ± 2	72 ± 3	0.58
Nadir, %	47 ± 4	49 ± 5	0.62
Peak, %	76 ± 2	76 ± 1	0.12
Recovery, %	73 ± 2	74 ± 3	0.97
Occlusion, AUC	1.74E+05 ± 8.92E+03	1.77E+05 ± 1.07E+04	0.61
Reperfusion, AUC	1.29E+05 ± 3.75E+03	1.30E+05 ± 4.43E+03	0.93

Post intervention	PS	IPC	Condition	Time	Interaction
Baseline, %	74 ± 1	75 ± 3	0.07	**<0.001**	0.25
Nadir, %	47 ± 3	50 ± 6	0.16	0.06	0.12
Peak, %	77 ± 1	78 ± 1	**0.04**	**<0.001**	0.24
Recovery, %	73 ± 1	74 ± 3	0.68	0.62	0.74
Occlusion, AUC	1.78E+05 ± 5.60E+03	1.81E+05 ± 9.50E+03	0.47	**0.007**	0.80
Reperfusion, AUC	1.44E+05 ± 2.10E+03	1.34E+05 ± 3.87E+03	0.22	0.11	0.37

*Note:* Bold values indicates *p* < 0.05.

**FIGURE 3:**
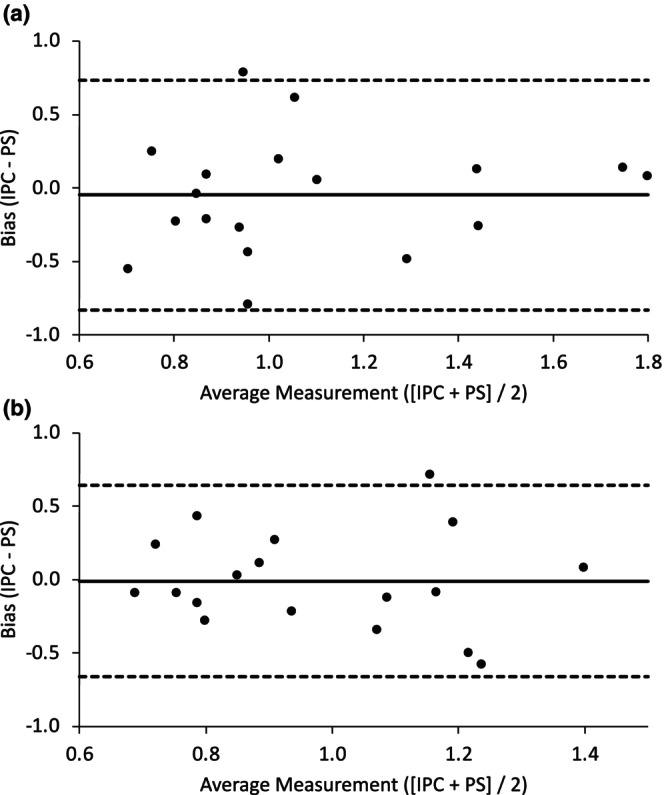
(a) Bland–Altman plot for resaturation rates between conditions. (b) Bland–Altman plot for rate constants between conditions. Groups include nine females and nine males.

The Bland–Altman plot suggested good agreement and minimal bias for the reperfusion rate (Figure 3a), while the ICC indicated significant reliability for the absolute agreement between PS and IPC resaturation rates (*r* = 0.47 [95% CI: 0.02, 0.76], *p* = 0.02). Further, while there was good agreement and minimal bias between procedures for oxidative capacity, as shown with the Bland–Altman analysis (Figure 3b), the ICC values were no longer in agreement (*r* = 0.25 [−0.26, 0.64], *p* = 0.16).

## 4 DISCUSSION

This study finds PS as an effective method to enhance microvascular reactivity and skeletal muscle oxidative capacity, offering complementary benefits at both the microcirculatory and muscular levels. Specifically, our findings demonstrate that a single bout of PS increases the microvascular desaturation and resaturation rate, which was like IPC, while also increasing skeletal muscle oxidative capacity. These results suggest that PS holds promise as a non‐pharmacological intervention to simultaneously increase vascular and muscle oxidative function.

### 4.1 Near‐infrared spectroscopy

Both PS and IPC led to steeper NIRS‐derived desaturation rates, which suggest a faster metabolism after these treatments (Barstow, 2019; Butenas et al., 2023). We are the first to show this after PS; however, the higher local metabolic rate contrasts with studies using acute and chronic IPC, which report a slower metabolic rate (Ambrozic et al., 2013; Jeffries et al., 2018). We believe this may be due to methodological differences in the muscle group studied (e.g., thenar eminence (Ambrozic et al., 2013)) and differences in fiber composition (Edgerton et al., 1975; Jeffries et al., 2018; Moore et al., 2021). Whether muscle‐specific responses to PS are influenced by fiber type composition, habitual activity levels, or training status requires further work. Understanding these differences may help optimize stretching protocols for enhancing muscle and metabolic function.

Microvascular resaturation rates increased after both PS and IPC, suggesting improved vascular responsiveness. However, neither protocol elicited a significantly better effect at the condition level, and the ICC and Bland–Altman analysis further suggest the improved microvascular resaturation rates were similar between interventions. These comparable increases in resaturation rates are noteworthy when considering the stimuli between interventions. IPC uses vascular occlusion to reduce flow, while PS causes mechanical tissue deformation and reduces blood flow (Caldwell et al., 2023; Mathieu‐Costello, 1987). These stimuli have been linked to increases in shear stress (Kruse et al., 2016), circulating nitrite (Rassaf et al., 2014), and an increase in prostacyclin levels (Rytter et al., 2020). It is also interesting to note that long‐term outcomes appear to be more favorable for PS, as a variety of local and systemic cardiovascular measures are improved (e.g., decreased vascular stiffness and increases in dilatory capacity) (Bisconti et al., 2020; Ce et al., 2022; Hotta et al., 2019), while evidence with IPC appears to be inconsistent (Enko et al., 2011; Heusch & Rassaf, 2016; Jeffries et al., 2018). Taken together, acute PS and IPC are beneficial for NIRS‐derived outcomes, and we hope that future work will further our mechanistic understanding of the muscle and vascular improvements after PS.

### 4.2 Muscle oxidative capacity

This is the first study to demonstrate that acute intermittent PS increases muscle oxidative capacity in healthy college‐aged males and females. Although PS was not superior to IPC, growing evidence suggests using PS is a low‐intensity method to enhance vascular resiliency (Caldwell et al., 2023), reduce arterial stiffness (Yamato et al., 2017), and increase glucose uptake (Kerris et al., 2019). The observed increase in muscle oxidative capacity following PS is particularly noteworthy, as it suggests a potential role for PS in improving metabolic and cardiovascular health. Given that muscle oxidative capacity is a key determinant of exercise performance, these findings have important implications for individuals with limited mobility or conditions such as peripheral arterial disease. While the underlying mechanisms remain unclear, they are likely linked to either shear‐ stress or stretch‐related receptors and their associated signaling pathways (Hotta et al., 2018; Jones et al., 2014).

### 4.3 Methodological considerations

While the present study was crossover in nature, there was no control intervention to compare the absence of stimuli to that of PS and IPC interventions. However, this investigation compared PS to IPC and the design reflects that. Moreover, this NIRS procedure does show good reliability indicating an unlikely change with a sham control (Gomez et al., 2008; Iannetta et al., 2019). The PS protocol was 5 min, and while there is no reported optimal time frame for PS, this study aimed to match the traditional 5‐min IPC protocol. A notable restriction was participant adipose tissue thickness surrounding the gastrocnemius as the NIRS device is half the source director distance, and we excluded two participants with greater than 1.0 cm of adipose tissue. The lack of reliability in the oxidative capacity test may be due to removal of data points for curve fitting and should be considered in the interpretation of data.

## 5 SUMMARY

In conclusion, these data highlight PS as a practical method to increase microvascular responsiveness and muscle oxidative capacity. Similar improvements between PS and IPC in microvascular function allude to the validity of PS as a useful method when physical exercise is limited. While there is much left to learn regarding precise mechanisms eliciting these microvascular responses, this study is a step forward to bridge the gap between our current insight and applied outcomes in which PS acts as a positive physiological stimulus.

## FUNDING INFORMATION

No additional funding.

## CONFLICT OF INTEREST STATEMENT

No conflicts of interest, financial or otherwise, are declared by the authors.

## ETHICS STATEMENT

All procedures involving human participants were approved by the University of Wisconsin‐La Crosse Institutional Review Board and conducted in accordance with the ethical standards of the Declaration of Helsinki. Written informed consent was obtained from all participants prior to their inclusion in the study.

## Data Availability

Data will be made available upon reasonable request.
